# Exploring the causal relationship between immune cell characteristics and melanoma: A two-way Mendelian randomization study

**DOI:** 10.1097/MD.0000000000042888

**Published:** 2025-06-13

**Authors:** Shanshan Wang, Hailong Xing

**Affiliations:** a Department of Dermatovenereology, Binzhou Central Hospital, Binzhou, Shandong, China; b Department of Neurosurgery, Binzhou Medical University Hospital, Binzhou, Shandong, China.

**Keywords:** causal reasoning, immunity, melanoma skin cancer, Mendelian randomized analysis

## Abstract

Melanoma skin cancer occurs mostly in melanocytes, with a high degree of malignancy and poor prognosis. The continuous development of Mendelian stochastic analysis in the field of medicine has led to the discovery of new therapeutic genes and drug targets for many diseases. Therefore, we aim to discover more possible melanoma therapeutic targets through Mendelian randomization analysis. Based on the published genome-wide association study data, 2-sample MR was used to analyze the 2-way causal relationship between 731 immune cell characteristics and melanoma skin cancer. The results of the inverse variance weighted method were used as the main screening criteria to obtain the characteristics of immune cells associated with melanoma. Heterogeneity analysis, multiplicity analysis and one-to-one elimination test also ensure the robustness and sensitivity of the results. We obtained 26 related immune cell characteristics, of which 15 immune cell characteristics are protective factors for melanoma. Including CD25 on transitional B cell, switched memory B cell, IgD+ CD38− B cell, BAFF-R on transitional B cell, CD38 on IgD+ CD38dim B cell, CD24+ CD27+ B cell, CD39+ activated CD4 regulatory T cell, BAFF-R on IgD− CD38− B cell, BAFF-R on IgD+ CD38+ B cell, CD28 on CD4 regulatory T cell, CD66b on CD66b++ myeloid cell, CD25 on CD24+ CD27+ B cell, BAFF-R on switched memory B cell, CD11b on granulocytic myeloid-derived suppressor cells and CD4RA on terminally differentiated CD4+ T cell. 11 immune cell characteristics were risk factors, including CD14+ CD16− monocyte, CD25++ CD45RA− CD4 not regulatory T cell absolute count, activated CD4 regulatory T cell absolute count, CD39+ CD8+ T cell absolute count, CCR2 on myeloid dendritic cell, CD127 on CD45RA+ CD4+ T cell, CD11c+ monocyte, CD3 on central memory CD8+ T cell, CD4+/CD8+ T cell, CD3 on HLA DR+ CD4+ T cell and CD4− CD8− T cell. Reverse analysis showed that melanoma had no effect on immune cell characteristics. Our study used MR method to analyze the causal relationship between 731 immune cell characteristics and melanoma from a genetic point of view, which provides more possible ways for the treatment of melanoma patients.

## 1. Introduction

Melanoma is a kind of malignant tumor in the skin system of cumulative patients. on the one hand, the malignant degree of melanoma is extremely high, and the prognosis and survival rate of patients are poor; on the other hand, patients in the early and middle stages have a 100% chance of developing into advanced patients. As long as distant metastasis occurs, the condition deteriorates rapidly, and the cycle from metastasis to death is only a few months.^[1,2]^ These factors make melanoma very tricky.

At present, the treatment of melanoma has been more mature, and the treatment of melanoma usually includes surgical resection, radiotherapy, chemotherapy, immunotherapy, and other methods. The specific treatment plan will be determined according to factors such as the patient’s condition, the size and location of the tumor and metastasis. Surgical resection is usually the 1st choice for early melanoma. The tumor is completely removed by surgery to prevent it from continuing to grow and spread.^[3,4]^ Radiotherapy can be used to treat unresectable tumors or to prevent the recurrence of melanoma. Although the therapeutic effect of chemotherapy for melanoma is limited, it can be used as an adjuvant therapy in some cases.^[5,6]^ Immunotherapy is a new type of treatment in recent years, and many studies have revealed many biomarkers related to cancer immunotherapy,^[7,8]^ so immune checkpoint inhibitors such as anti-PD-1 and anti-CTLA-4 antibodies have been developed. These drugs can activate the body’s immune system and enhance the ability to recognize and kill melanoma cells.^[9,10]^ In addition, there are targeted therapy and some new treatments, which greatly improve the prognosis of patients with melanoma.^[11–13]^ However, long-term use of immunity and targeted therapy leads to treatment resistance in patients, which greatly reduces the efficacy. Therefore, it is a top priority to explore more possible treatments and targets for melanoma.

Mendelian randomization (MR) is a statistical design method with high statistical efficiency. Genetic information is used as a tool variable, and the correlation is not affected by causal inversion or confounding factors.^[14,15]^ Previous studies have reported the effects of about 22 million variants on the characteristics of 731 immune cells in 3757 Sardinians, and found complex genetic regulation of immune cells. These results determine the pathway of targeted drugs and provide a basis for the design of specific treatments for autoimmune diseases.^[16]^ Many previous studies have revealed that many immune cell characteristics are related to the occurrence, development and prognosis of melanoma, but there is no systematic genetic analysis of the causal relationship between them. Therefore, from the point of view of double-sample bidirectional MR, this study explores the causal relationship between immune cell characteristics and melanoma, and looks for potential therapeutic targets for melanoma.

## 2. Materials and methods

### 2.1. Genome-wide association study (GWAS) data acquisition

The genome-wide association study (GWAS) data for melanoma come from UKBiobank. The study included 3,75,767 European individuals, including 3751 melanoma patients and 3,72,016 controls, and received a total of 1,13,96,019 single nucleotide polymorphisms. The data of 731 immune cell characteristics were obtained from the public catalogue of GWAS database. The study analyzed the effects of about 22 million variants on the characteristics of 731 immune cells in 3757 Sardinese, the GWAS ID numbers are GCST90001391 to GCST90002121. Among the 731 immune cell characteristics, 118 absolute cell numbers, 389 surface antigens, 32 morphological parameters and 192 relative counts (the ratio between cell levels) were included.

### 2.2. Selection of instrumental variables (IVs)

In the MR study, we used a 2-sample MR method to assess the causal relationship between immune cell characteristics and melanoma skin cancer. Because too few genome-wide significant sites were screened according to the ideal threshold (5e−8), which may lead to low statistical power and weak instrumental variables (IVs), thus affecting the accuracy of parameter estimation, we adjusted the threshold to 1e−5 according to previous studies to ensure sufficient IVs for subsequent analysis. For melanoma skin cancer, we used the 5e−8 threshold again and obtained a total of 1121 single nucleotide polymorphisms that were significantly associated.

### 2.3. Linkage disequilibrium and removal of weak instrumental variables

For immune cell characteristics and GWAS data of melanin skin cancer, we use the default parameter, that is, clump = TRUE, *r*^2^ = 0.001, kb = 10,000. Then we remove weak IVs according to the screening criteria of *F* test value > 10, and filter out IVs that do not have a strong correlation with exposure factors or can only explain a small number of phenotypic variables. finally, we obtained 11 IVs for melanin skin cancer.

### 2.4. Mendelian randomized analysis

Then we used TwoSampleMR package to conduct a 2-way Mendelian randomized analysis between immune cell characteristics and melanoma skin cancer. Inverse variance weighted (IVW), weighted median, MR-Egger, simple mode and weighted mode were mainly used for analysis. We mainly used the standard of IVW *P* < .05 to screen the positive results.^[17,18]^ Because the tool variables from different analysis platforms, experiments and populations may have heterogeneity, which affects the results of Mendelian randomized analysis, we evaluate the heterogeneity by IVW and MR-Egger test. *P* value < .05 indicates that there is heterogeneity in the study. If there is no heterogeneity and pleiotropy, we choose IVW method to screen the positive results; if there is heterogeneity, we screen the results according to the results of weighted median method. At the same time, we use MR-Egger intercept test to detect the multiplicity of data and evaluate the robustness of the results. If *P* value < . 05, it means that the data have multiple effects and should be removed. We use 1-by-1 elimination test (leave-one-out sensitivity test) to determine whether the results are sensitive or not.

All the statistical analyses are carried out in R4.3.1 software (https://www.r-project.org/), and some of the results are shown in graphic form.

## 3. Result

### 3.1. Exploring the impact of immune cell characteristics on the onset of melanoma skin cancer

**Figure 1.**
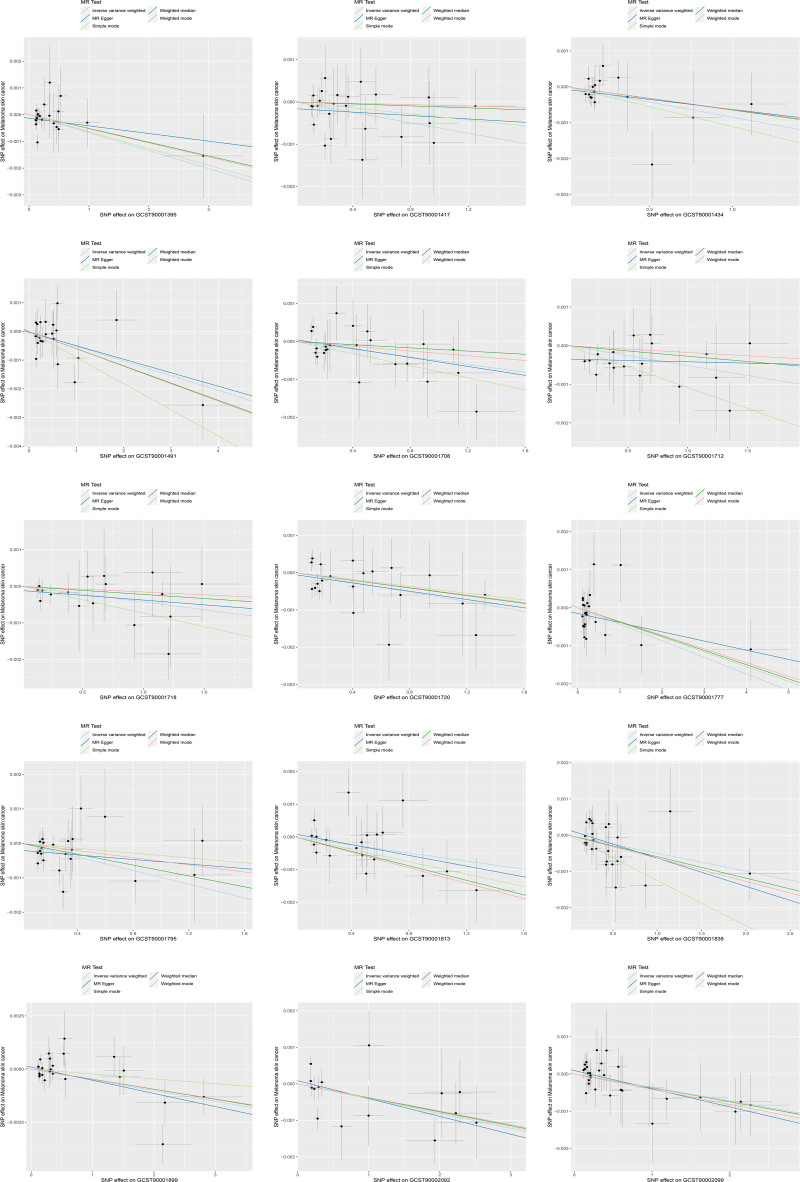
The impact of protective immune cell characteristics on the onset of melanoma skin cancer. MR = Mendelian randomization, SNP = single nucleotide polymorphism.

**Figure 2.**
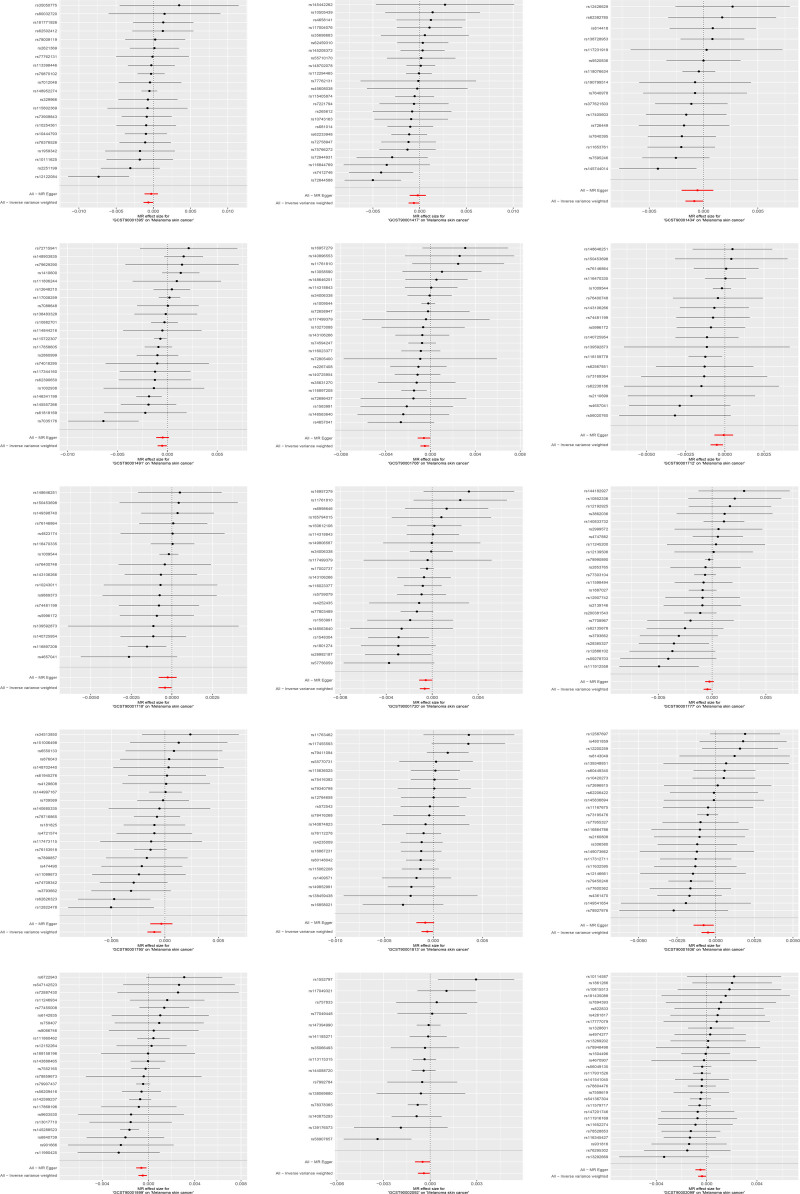
The forest map of protective immune cell characteristics and melanoma skin cancer risk. MR = Mendelian randomization.

**Figure 3.**
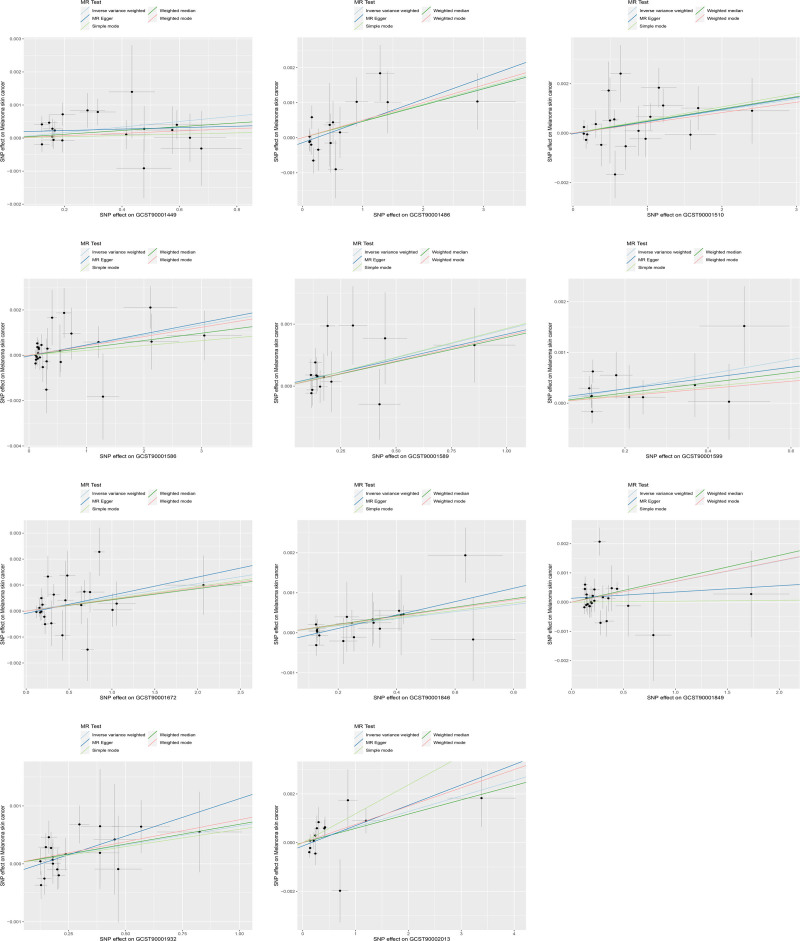
The impact of risk immune cell characteristics on the onset of melanoma skin cancer. MR = Mendelian randomization, SNP = single nucleotide polymorphism.

**Figure 4.**
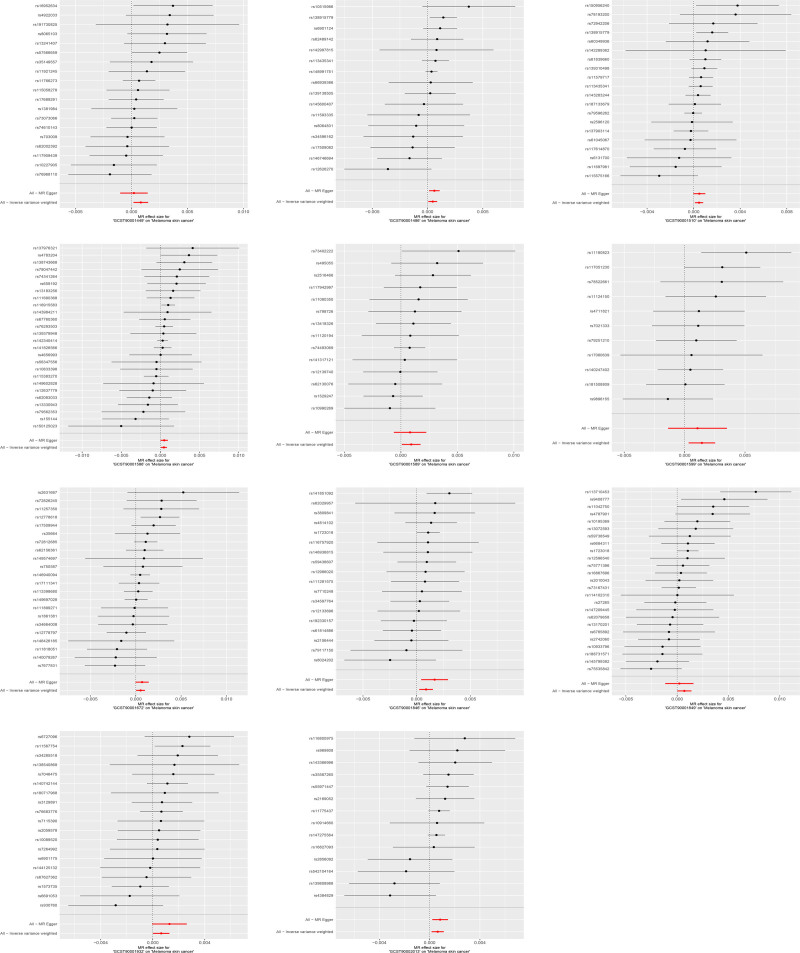
The forest map of risk immune cell characteristics and melanoma skin cancer risk. MR = Mendelian randomization.

Using IVW as the main method, we screened 26 immune cell characteristics related to melanoma skin cancer, including 10 B cell related characteristics, 11 T cell related characteristics, 2 monocyte related characteristics and 3 myeloid cell related characteristics (Table 1). Among these related immune cell characteristics, 15 features showed protective effect on melanoma skin cancer. Includes CD25 on transitional B cell, Switched memory B cell, IgD+ CD38− B cell, BAFF-R on transitional B cell, CD38 on IgD+ CD38dim B cell, CD24+ CD27+ B cell, CD39+ activated CD4 regulatory T cell, BAFF-R on IgD− CD38− B cell, BAFF-R on IgD+ CD38+ B cell, CD28 on CD4 regulatory T cell, CD66b on CD66b++ myeloid cell, CD25 on CD24+ CD27+ B cell, BAFF-R on switched memory B cell, CD11b on granulocytic myeloid-derived suppressor cells and CD4RA on terminally differentiated CD4+ T cell (Table 2 and Figs. 1 and 2). The other 11 features are risk factors for melanoma skin cancer, including CD14+ CD16− monocyte, CD25++ CD45RA− CD4 not regulatory T cell absolute count, activated CD4 regulatory T cell absolute count, CD39+ CD8+ T cell absolute count, CCR2 on myeloid dendritic cell, CD127 on CD45RA+ CD4+ T cell, CD11c+ monocyte, CD3 on central memory CD8+ T cell, CD4+/CD8+ T cell, CD3 on HLA DR+ CD4+ T cell and CD4-CD8− T cell (Table 3 and Figs. 3 and 4). Heterogeneity analysis, multiplicity analysis and 1-by-1 elimination test all proved the robustness of the results (Tables S1 and S2, Supplemental Digital Content, https://links.lww.com/MD/P187 and Figs. S1–S4, Supplemental Digital Content, https://links.lww.com/MD/P188).

**Table 1:** The characteristics of 731 immune cells in the GWAS database.

GWAS ID	Traits
GCST90001395	IgD+ CD38− B cell %B cell
GCST90001417	CD24+ CD27+ B cell %B cell
GCST90001434	Switched memory B cell %lymphocyte
GCST90001449	CD11c+ monocyte %monocyte
GCST90001486	Activated CD4 regulatory T cell absolute count
GCST90001491	CD39+ activated CD4 regulatory T cell %CD4 regulatory T cell
GCST90001510	CD25++ CD45RA− CD4 not regulatory T cell absolute count
GCST90001586	CD14+ CD16− monocyte %monocyte
GCST90001589	CD4+/CD8+ T cell
GCST90001599	CD4-CD8− T cell %T cell
GCST90001672	CD39+ CD8+ T cell absolute count
GCST90001708	BAFF-R on IgD+ CD38+ B cell
GCST90001712	BAFF-R on IgD− CD38− B cell
GCST90001718	BAFF-R on switched memory B cell
GCST90001720	BAFF-R on transitional B cell
GCST90001777	CD25 on CD24+ CD27+ B cell
GCST90001795	CD25 on transitional B cell
GCST90001813	CD38 on IgD+ CD38dim B cell
GCST90001836	CD66b on CD66b++ myeloid cell
GCST90001846	CD3 on central memory CD8+ T cell
GCST90001849	CD3 on HLA DR+ CD4+ T cell
GCST90001899	CD28 on CD4 regulatory T cell
GCST90001932	CD127 on CD45RA+ CD4+ T cell
GCST90002013	CCR2 on myeloid dendritic Cell
GCST90002092	CD11b on granulocytic myeloid-derived suppressor cells
GCST90002099	CD4RA on terminally differentiated CD4+ T cell

BAFF = B-cell activating factor of the TNF family, BAFF-R = B-cell activating factor receptor, GWAS = genome-wide association study, HLA = human leukocyte antigen, IgD = immunoglobulin D.

**Table 2:** The impact of immune cell characteristics on the onset of melanoma skin cancer (protective immune cell characteristics).

Outcome	Exposure	*P*val_IVW	OR	or_lci95	or_uci95
Melanoma skin cancer	GCST90001395	.04272	0.99933	0.99868	0.99998
Melanoma skin cancer	GCST90001417	.03444	0.99938	0.99881	0.99995
Melanoma skin cancer	GCST90001434	.04258	0.99915	0.99834	0.99997
Melanoma skin cancer	GCST90001491	.02892	0.99949	0.99902	0.99995
Melanoma skin cancer	GCST90001708	.01216	0.99949	0.99910	0.99989
Melanoma skin cancer	GCST90001712	.01146	0.99949	0.99909	0.99988
Melanoma skin cancer	GCST90001718	.03716	0.99957	0.99917	0.99997
Melanoma skin cancer	GCST90001720	.00317	0.99935	0.99892	0.99978
Melanoma skin cancer	GCST90001777	.01635	0.99957	0.99921	0.99992
Melanoma skin cancer	GCST90001795	.00265	0.99901	0.99837	0.99966
Melanoma skin cancer	GCST90001813	.03737	0.99938	0.99880	0.99996
Melanoma skin cancer	GCST90001836	.01944	0.99950	0.99909	0.99992
Melanoma skin cancer	GCST90001899	.00726	0.99950	0.99913	0.99986
Melanoma skin cancer	GCST90002092	.03614	0.99959	0.99921	0.99997
Melanoma skin cancer	GCST90002099	.02435	0.99964	0.99933	0.99995

OR = odds ratio, or_lci95 = lower 95% confidence interval of the odds ratio, or_uci95 = upper 95% confidence interval of the odds ratio, *P*val_IVW = *P* value inverse variance weighted.

**Table 3:** The impact of immune cell characteristics on the onset of melanoma skin cancer (risk immune cell characteristics).

Outcome	Exposure	*P*val_IVW	OR	or_lci95	or_uci95
Melanoma skin cancer	GCST90001449	.01281	1.00084	1.00018	1.00149
Melanoma skin cancer	GCST90001486	.02244	1.00047	1.00007	1.00087
Melanoma skin cancer	GCST90001510	.01138	1.00046	1.00010	1.00082
Melanoma skin cancer	GCST90001586	.02542	1.00044	1.00005	1.00084
Melanoma skin cancer	GCST90001589	.02994	1.00092	1.00009	1.00174
Melanoma skin cancer	GCST90001599	.01074	1.00143	1.00033	1.00253
Melanoma skin cancer	GCST90001672	.03277	1.00053	1.00004	1.00102
Melanoma skin cancer	GCST90001846	.00703	1.00088	1.00024	1.00152
Melanoma skin cancer	GCST90001849	.03600	1.00070	1.00001	1.00140
Melanoma skin cancer	GCST90001932	.04263	1.00065	1.00002	1.00129
Melanoma skin cancer	GCST90002013	.01029	1.00064	1.00015	1.00113

OR = odds ratio, or_lci95 = lower 95% confidence interval of the odds ratio, or_uci95 = upper 95% confidence interval of the odds ratio, *P*val_IVW = *P* value inverse variance weighted.

### 3.2. Exploring the impact of melanoma skin cancer on immune cell signatures

We also carried out reverse causality analysis, using double-sample MR analysis and IVW method as the main analysis method. The results showed that the *P* values were all >.05. The results were not statistically significant (Table S3, Supplemental Digital Content, https://links.lww.com/MD/P187).

## 4. Discussion

Melanoma skin cancer is a highly malignant tumor with rapid progression and can rapidly grow and spread to other parts in a short time, seriously affecting the quality of life and prognosis of patients. At present, the incidence of melanoma is increasing year by year, and it has become one of the main causes of skin cancer death worldwide. Therefore, the treatment of melanoma skin cancer is an important clinical problem.

The treatment of melanoma includes surgical resection, radiotherapy, chemotherapy, immunotherapy and so on. Previous studies have shown that melanoma is a kind of immunogenic tumor, so immunotherapy is an effective means to treat melanoma. Its core idea is to activate or enhance the body’s immune system, so that it can more effectively identify, attack and eliminate disease-related abnormal cells, including cancer cells and pathogenic microorganisms of infectious diseases. It has many advantages: personalized treatment, long-lasting therapeutic effect and relatively few side effects. At present, immunotherapy for patients with melanoma has immunocheckpoint inhibitors, including CTLA-4 inhibitors and PD-1/PD-L1 inhibitors, which is one of the main immunotherapies used to treat melanoma. Chimeric antigen receptor T cell therapy is a new type of immunotherapy, which can better recognize and attack melanoma cells by transforming patients’ own T cells. Cancer vaccines: cancer vaccines against melanoma have shown some potential in clinical trials, including personalized cancer vaccines and specific antigen vaccines. However, there are also some challenges, such as low efficiency for some patients, many side effects, expensive treatment and so on. Therefore, it is necessary to find a more suitable and safer therapeutic target.

MR is a commonly used experimental design method, whose core is to use genetic data as a bridge to explore the causal relationship between an exposure and an outcome. Because genetic variation is born with birth, the relationship between genetic variation and outcome is in accordance with causal time sequence, and will not be disturbed by other factors, which can better help researchers to determine the direction of causality, so as to solve the common confounding and reverse causal problems in observational studies, and draw conclusions closer to causality. Therefore, the use of MR method for causal inference of observational research has become a hot topic at present.^[19,20]^ Therefore, in this study, we used MR method to explore the causal relationship between 731 immune cell characteristics and melanoma skin cancer, and to find possible therapeutic targets.

In this study, based on the genetic data of large sample size, we found a causal relationship between 26 immune cell characteristics and melanoma. Previous studies have shown that the relative frequency of memory B cells is higher in patients with melanoma. Compared with the circulation of melanoma patients, the ratio of IgD− memory to initial B cells in the tumor increased, indicating that the humoral response in tumor microenvironment tends to be type-switching.^[21]^ In addition, BAFF is related to the production of reactive IgG in tumor cells of melanoma patients.^[22]^ Some studies have also shown that BAFF may be a serum marker related to uveal melanoma metastasis,^[23]^ and BAFF is also a key driver of anti-tumor immune response induced by dendritic cells in melanoma host.^[24]^ CD39 is an important immunosuppressive molecule. It can degrade adenosine triphosphate and reduce the level of adenosine triphosphate in tumor microenvironment, thus inhibit the activity of T cells and natural killer cells. The expression of CD39 on regulatory T cells inhibits the activity of natural killer cell, which is related to liver metastasis and may be a carcinogenic factor, which is also reflected in our study.^[25]^ CD66b is mostly expressed in neutrophils and plays an important role in inflammation and immune response. Studies have shown that the infiltration of CD66b neutrophils and CD123 dendritic cells in primary melanoma is independently related to poor prognosis.^[26]^ The number of CD14−CD66b+arginase+tumor-related granular myeloid inhibitory cells in peripheral blood mononuclear cells of patients with metastatic melanoma increased, which inhibited the proliferation of autologous T cells.^[27]^ Other studies have found that CD39 + CD8 + T cells exist in tumors and invaded or metastatic lymph nodes in breast cancer and melanoma patients, which is related to the occurrence and development of breast cancer and melanoma.^[28]^ Carsten Krieg and Rahul Chavan et al found that the frequency of classic CD14 + CD16-HLA-DRhi monocytes can predict the response of melanoma to anti-PD-1 immunotherapy and is associated with immunosuppression of melanoma, thus helping to stratify patients before immunotherapy.^[29,30]^ CCR2 is a chemokine receptor. Some studies have shown that CCR2 may affect the development of melanoma by regulating immune cell migration and inflammatory response.^[31,32]^ Sabino Strippoli et al have shown that circulating CD4−/CD8− double negative T cells change during checkpoint inhibitor therapy and may be good at sensing the immune response to melanoma. The complementary variation of circulating CD4/CD8− double negative T cells relative to CD4+ and other immune factors may improve the reliability of lymphocyte evaluation and is expected to become a potential therapeutic target for melanoma.^[33]^ Our study also found that CD4−CD8− Tcell is a risk factor for melanoma. In addition, a variety of causal immune cell characteristics obtained in this study have not been revealed, but they may also be potential therapeutic targets for patients with melanoma.

In this study, MR was used to systematically analyze the GWAS data of melanoma patients and 731 types of immune cells. Compared with previous studies, this study has significant innovations in methodology and research design. First, this study adopted MR, using genetic variation as an IV to effectively reduce the influence of confounding factors, so as to more accurately infer the causal association between melanoma and immune cells. Compared with traditional observational studies, this method can provide more reliable evidence for causal inference. Secondly, this study is based on large-scale GWAS data, with a large sample size, and the statistical validity and reliability of the results are higher, providing a more stable reference for research in the field. The sample size is large, the results are convincing, and the sensitivity analysis ensures the reliability of the conclusions. This study corroborates previous studies and provides more evidence from a genetic perspective. The findings provide a new direction for melanoma treatment strategies. By identifying genetic variants associated with immune cells, it can provide a basis for the development of new therapeutic targets. For example, if certain genetic variants are strongly linked to the activity or function of specific immune cells, treatments that target those variants may help boost the immune system’s ability to attack tumors. In addition, these genetic variants can be used as biomarkers to predict a patient’s response to immunotherapy, enabling personalized treatment. However, this study also has certain limitations that need to be discussed in more depth. First, the study’s database is based on European populations, and there is no data from other ethnic groups to analyze, so the generalizability of the conclusions is limited. There are differences in genetic background and environmental factors between different ethnic groups, which may make the findings less applicable to other populations. Future studies should consider including more ethnically diverse data to improve the generality of the findings. Secondly, this study adjusted the threshold from the ideal 5e−8 to 1e−5 in the selection of IVs to obtain more significant sites. While this adjustment increases the statistical power of the analysis, it can also lead to weaker correlations between IVs and exposure factors, introducing bias. In addition, weak IVs (*F*-test values < 10) may not effectively account for variation in exposure factors, further affecting the accuracy of parameter estimates. In addition, pleiotropy is also an issue to consider, in which a single gene may affect more than 1 phenotype, which can complicate the inference of causality. Finally, although randomization can reduce the influence of confounding factors, it cannot completely eliminate individual differences. In the actual process, there may still be some uncontrollable individual differences that affect the comparability of experimental results. For example, factors such as an individual’s lifestyle and environmental exposure may have an impact on the findings.

In summary, this study has some value in providing evidence of a genetic link between melanoma and immune cells, but there are some limitations. Future studies should focus on the potential bias, the possibility of pleiotropy, and the selection of genetic variants to further improve the validity of the study and the reliability of the conclusions. After refining the GWAS data, a comprehensive and systematic multi-ethnic analysis is necessary, which will contribute to a more complete understanding of the genetic mechanisms of melanoma and provide more targeted strategies for the prevention and treatment of the disease.

## 5. Conclusion

In conclusion, we used the MR method to conduct a bidirectional analysis of the causal relationship between melanoma and immune cell characteristics from the perspective of genetics, revealing the immune cell characteristics that may be related to the pathogenesis of melanoma, and providing ideas for the treatment of melanoma.

## Author contributions

**Data curation:** Shanshan Wang, Hailong Xing.

**Formal analysis:** Hailong Xing.

**Methodology:** Shanshan Wang.

**Software:** Hailong Xing.

**Writing – original draft:** Shanshan Wang.

**Writing – review & editing:** Hailong Xing.

## Supplementary Material


