# Supplementary material for: Exploring the causal relationship between immune cell characteristics and melanoma: A two-way Mendelian randomization study
Source: Medicine (Baltimore). 2025 Jun 13;104(24):e42888. doi: 10.1097/MD.0000000000042888 (PMC12173254; doi:10.1097/MD.0000000000042888)
Supplement: Supplementary file 1 [file medi-104-e42888-s001.pdf]

Supplementary Table 1 The impact of immune cell characteristics on the onset of melanoma skin cancer (Heterogeneity analysis and pleiotropy analysis- Protective immune cell characteristics)

outcome	exposure	Q_MR.Egger	Q_df_MR.Egger	Q_pval_MR.Egger	Q_Ivw	Q_df_Ivw	Q_pval_Ivw	egger intercept	se	pval
Melanoma skin cancer	GCST90001795	17.31477	20.00000	0.63245	19.87431	21.00000	0.52923	-0.00020	0.00013	0.12531
Melanoma skin cancer	GCST90001434	11.11204	14.00000	0.67721	11.33801	15.00000	0.72829	-0.00007	0.00015	0.64186
Melanoma skin cancer	GCST90001395	14.76721	19.00000	0.73727	16.22384	20.00000	0.70264	-0.00013	0.00011	0.24228
Melanoma skin cancer	GCST90001720	20.64709	19.00000	0.35662	20.89203	20.00000	0.40351	-0.00005	0.00012	0.64037
Melanoma skin cancer	GCST90001813	18.54341	18.00000	0.42043	18.89207	19.00000	0.46378	0.00009	0.00016	0.56794
Melanoma skin cancer	GCST90001417	20.38508	22.00000	0.55897	21.94916	23.00000	0.52332	-0.00016	0.00013	0.22421
Melanoma skin cancer	GCST90001491	31.21654	20.00000	0.05240	31.27870	21.00000	0.06914	-0.00003	0.00015	0.84383
Melanoma skin cancer	GCST90001712	7.00730	16.00000	0.97312	10.83510	17.00000	0.86501	-0.00035	0.00018	0.06810
Melanoma skin cancer	GCST90001708	16.96626	21.00000	0.71315	17.13635	22.00000	0.75579	0.00005	0.00012	0.68422
Melanoma skin cancer	GCST90001899	25.72194	22.00000	0.26381	26.70301	23.00000	0.26881	0.00009	0.00010	0.36958
Melanoma skin cancer	GCST90001836	19.99653	23.00000	0.64212	21.20838	24.00000	0.62640	0.00014	0.00013	0.28235
Melanoma skin cancer	GCST90001777	25.98271	22.00000	0.25243	29.60592	23.00000	0.16105	-0.00015	0.00009	0.09379
Melanoma skin cancer	GCST90001718	8.23993	15.00000	0.91380	8.98505	16.00000	0.91403	-0.00012	0.00014	0.40162
Melanoma skin cancer	GCST90002092	20.41969	13.00000	0.08523	20.88602	14.00000	0.10459	0.00009	0.00016	0.59507
Melanoma skin cancer	GCST90002099	12.82342	27.00000	0.99033	13.83928	28.00000	0.98829	0.00009	0.00009	0.32245

Supplementary Table 2 The impact of immune cell characteristics on the onset of melanoma skin cancer (Heterogeneity analysis and pleiotropy analysis- Risk immune cell characteristics)

outcome	exposure	Q_MR.Egger	Q_df_MR.Egger	Q_pval_MR.Egger	Q_Ivw	Q_df_Ivw	Q_pval_Ivw	egger intercept	se	pval
Melanoma skin cancer	GCST90001586	22.31480	24.00000	0.56048	22.42441	25.00000	0.61113	-0.00003	0.00010	0.74346
Melanoma skin cancer	GCST90001510	18.89199	18.00000	0.39850	18.91504	19.00000	0.46230	-0.00003	0.00017	0.88384
Melanoma skin cancer	GCST90001486	13.02567	14.00000	0.52450	14.31871	15.00000	0.50151	-0.00014	0.00012	0.27458
Melanoma skin cancer	GCST90001672	20.91315	20.00000	0.40226	21.31035	21.00000	0.44014	-0.00009	0.00014	0.54463
Melanoma skin cancer	GCST90002013	14.35962	12.00000	0.27834	15.50684	13.00000	0.27679	-0.00014	0.00014	0.34684
Melanoma skin cancer	GCST90001932	12.06337	17.00000	0.79628	13.19047	18.00000	0.78014	-0.00017	0.00016	0.30324
Melanoma skin cancer	GCST90001849	37.26439	23.00000	0.03050	38.31352	24.00000	0.03219	0.00013	0.00016	0.42924
Melanoma skin cancer	GCST90001449	14.68694	17.00000	0.61802	16.04205	18.00000	0.58961	0.00018	0.00016	0.26047
Melanoma skin cancer	GCST90001846	7.63540	16.00000	0.95899	9.72851	17.00000	0.91461	-0.00023	0.00016	0.16727
Melanoma skin cancer	GCST90001589	8.64569	12.00000	0.73286	8.67533	13.00000	0.79700	0.00003	0.00015	0.86619
Melanoma skin cancer	GCST90001599	8.95604	9.00000	0.44134	9.06206	10.00000	0.52622	0.00007	0.00021	0.75216

Supplementary Table 3 The impact of melanoma skin cancer on immune cell signatures

outcome	exposure	pval_Ivw
GCST90001395.rds	Melanoma skin cancer	0.81919
GCST90001417.rds	Melanoma skin cancer	0.84502
GCST90001434.rds	Melanoma skin cancer	0.94271
GCST90001449.rds	Melanoma skin cancer	0.74633
GCST90001486.rds	Melanoma skin cancer	0.35729
GCST90001491.rds	Melanoma skin cancer	0.37914
GCST90001510.rds	Melanoma skin cancer	0.28902
GCST90001586.rds	Melanoma skin cancer	0.39681
GCST90001589.rds	Melanoma skin cancer	0.32826
GCST90001599.rds	Melanoma skin cancer	0.99569
GCST90001672.rds	Melanoma skin cancer	0.16543
GCST90001708.rds	Melanoma skin cancer	0.27357
GCST90001712.rds	Melanoma skin cancer	0.09214
GCST90001718.rds	Melanoma skin cancer	0.11224
GCST90001720.rds	Melanoma skin cancer	0.43064
GCST90001777.rds	Melanoma skin cancer	0.20457
GCST90001795.rds	Melanoma skin cancer	0.69509
GCST90001813.rds	Melanoma skin cancer	0.25646
GCST90001836.rds	Melanoma skin cancer	0.94783
GCST90001846.rds	Melanoma skin cancer	0.08118
GCST90001849.rds	Melanoma skin cancer	0.96128
GCST90001899.rds	Melanoma skin cancer	0.36967
GCST90001932.rds	Melanoma skin cancer	0.72543
GCST90002013.rds	Melanoma skin cancer	0.76389
GCST90002092.rds	Melanoma skin cancer	0.41358
GCST90002099.rds	Melanoma skin cancer	0.46564
