# Supplementary material for: Exploring the causal relationship between immune cell characteristics and melanoma: A two-way Mendelian randomization study
Source: Medicine (Baltimore). 2025 Jun 13;104(24):e42888. doi: 10.1097/MD.0000000000042888 (PMC12173254; doi:10.1097/MD.0000000000042888)

Supplementary Figure 1 The Funnel plot of protective immune cell characteristics and melanoma skin cancer risk.

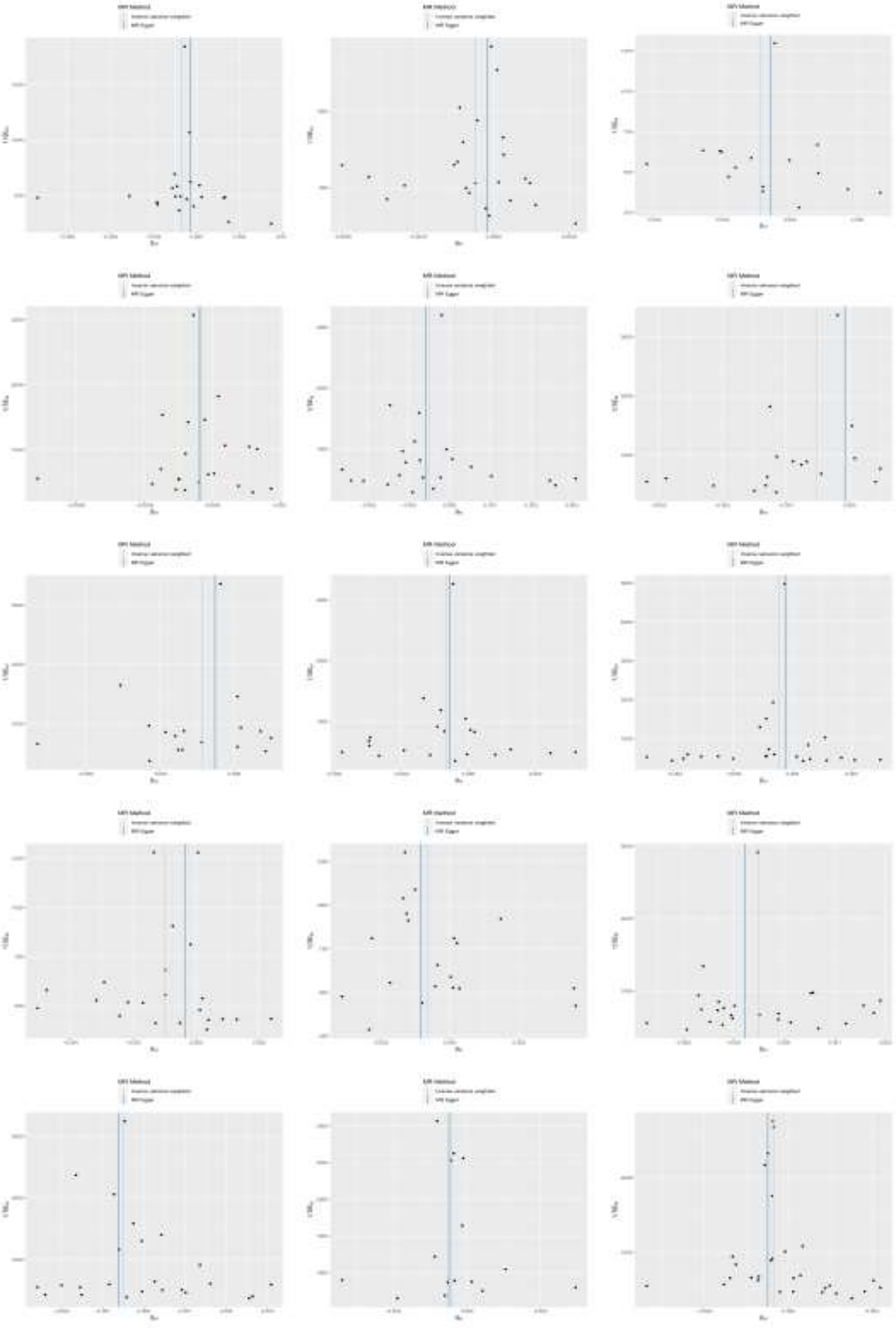

Supplementary Figure 2 The Funnel plot of risk immune cell characteristics and melanoma skin cancer risk.

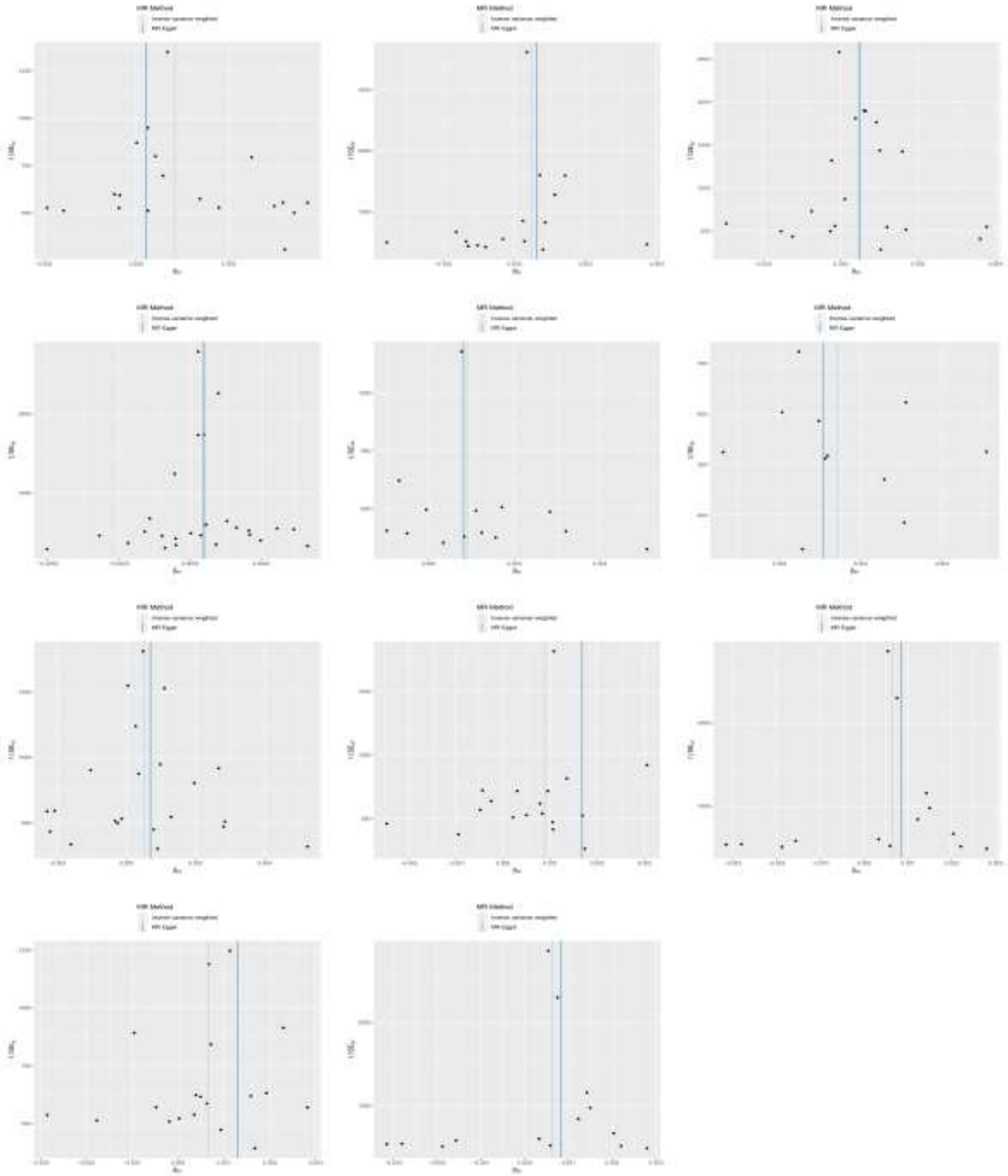

Supplementary Figure 3 The Sensitivity analysis of protective immune cell characteristics and melanoma skin cancer risk.

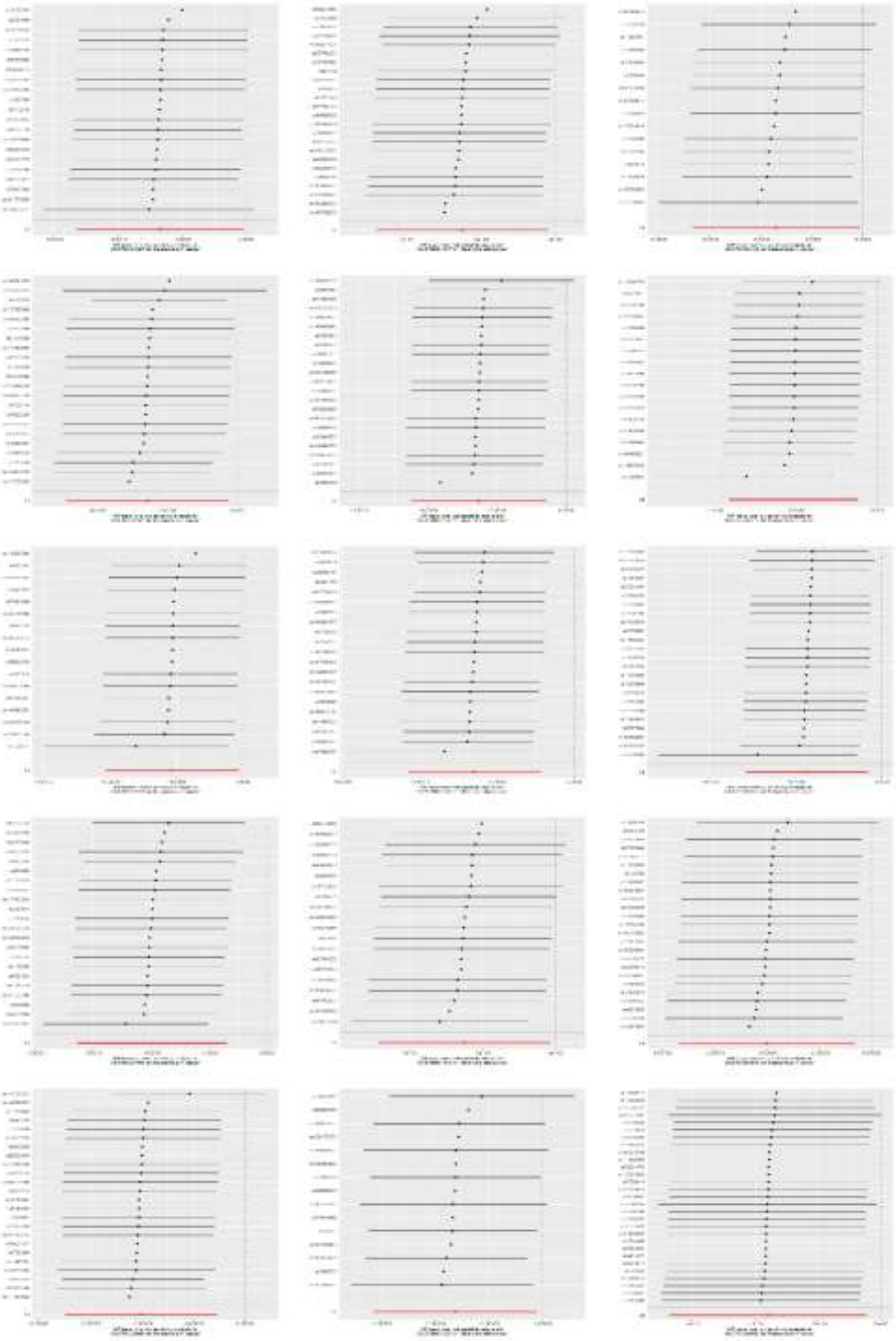

Supplementary Figure 4 The Sensitivity analysis of risk immune cell characteristics and melanoma skin cancer risk.

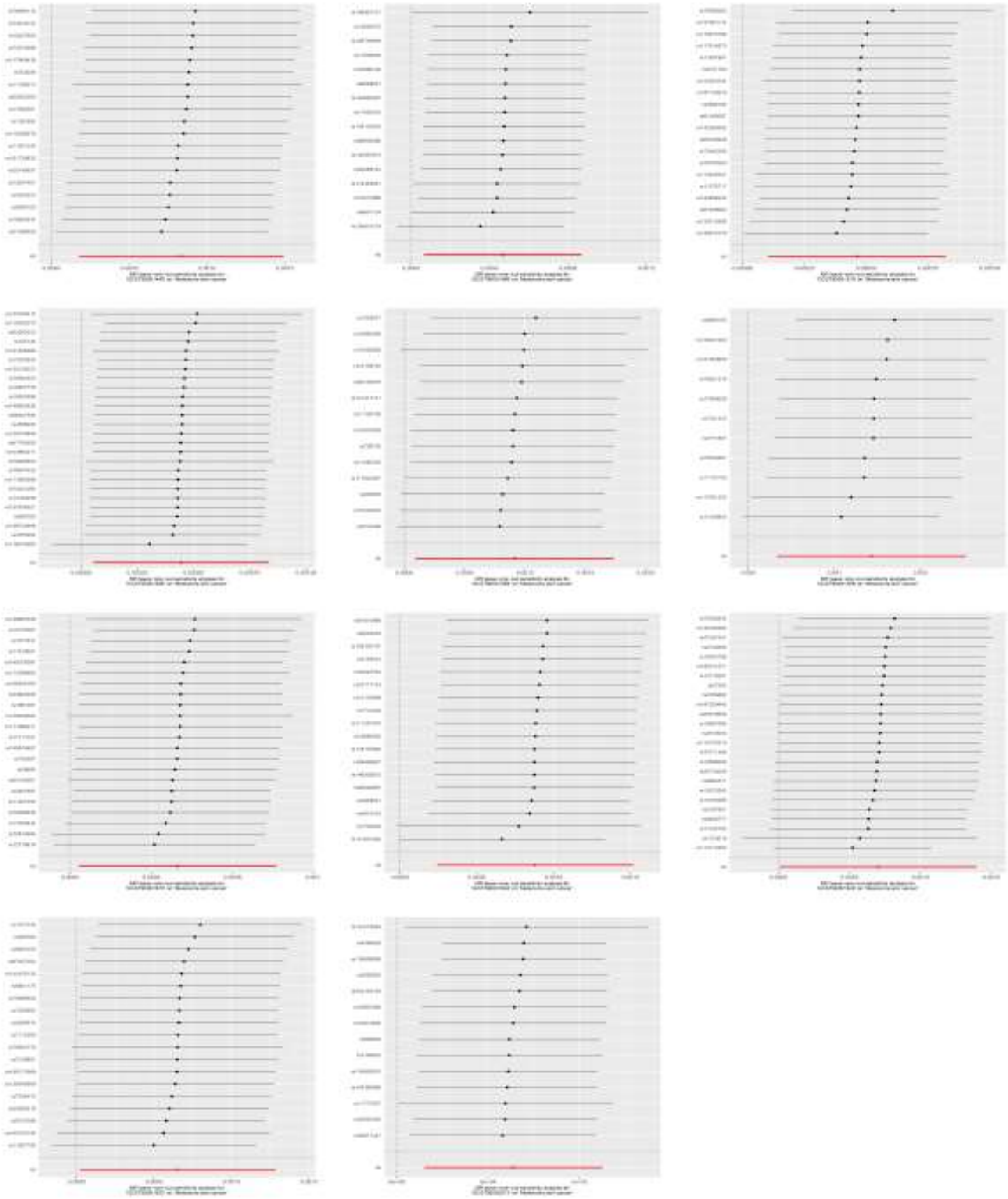

Supplement: Supplementary file 2 [file medi-104-e42888-s002.pdf]
